# Vent Filling

**Published:** 1858-06

**Authors:** W. H. Atkinson


### VENT FILLING.

BY W. H. ATKINSON.

Very early in my practice, (I should think as early as 1838,) it oftener occurred to the Dental Practitioner, to be called on to insert pivot teeth, than it now does. Having observed that pivot teeth, set upon fangs that had been dead, frequently swelled, and sometimes formed alveolar abscess, whilst those set upon fangs, with recently destroyed nerves, seldom, or almost never caused inconvenience.

Conceiving the inconvenience to result from pent up foreign matter, I was induced to make a channel on one side of the pivot, admitting the escape of gasses or fluid substances, and the result verified my suspicions to such an extent, that it passed into a rule of action invariably, to leave a vent in such cases.

Among my first efforts at fang filling, a portion of them gave me trouble, as had my early efforts at pivoting teeth, and conceiving a close analogy to exist, thought a vent would operate as well in this as in a former case—I tested it, and have always been satisfied with the results of the experiment.

### MODE OF OPERATION.

After having perfectly excavated the cavity, and shaped it to your mind, and rimmed out the fang as far up as possible, I take a piece of wire, of any tough metal, and after having drawn it to a firm point, and burnished smooth, dip it into creosote, and force it to the apex of the fang if possible. Fill the fang around the wire close and solid, being careful to preserve it in a direct line. Proceed to fill and finish the main cavity; then with a pair of flat-nosed plyers, withdraw the wire, thus leaving a small tube.

All that is necessary for teeth having more than one fang, is to add a wire for each fang.

### CASE.

Meadville, Pa., Sept. 1849.—Mr. A. H, requested pivot teeth set on the incisors and canines, of the superior maxillary.

Trimmed down, drilled holes, inserted on compressed hickory pivots, without obliterating the balance of dental canal. About the third day after the operation, patient called with face tremendously swelled, and in great pain. Made an attempt to remove the pivots according to the then authority, found it impracticable for fear of removing fang and all. Upon more minute inspection, observed fluctuation at the point of each fang, into which I plunged a lancet. Bloody matter flowed freely, giving almost instant relief. In fact I made the six abscesses, as it were, but one. A bottle of creosote being upon my table, by inspiration (for I never had heard of such a thing before) I wet a pleget of cotton in it, and swabbed out each abscess; in a few days it was all well, and teeth all firm in the sockets. Two years after patient left that town, but I learned in 1850, that the teeth were setting on same pivots, and doing good service.

NOTE.

This case opportunely occurring, taught me that ulcerated teeth might be cured, at least so far as single fanged teeth are concerned. Upon this early hint I have gradually improved, until I now conceive it practicable to save almost any tooth, as long as even a moiety of its periosteal connections remain firm.

Evidence of the above conclusion can be furnished by a series of cases, the history of which has been kept from the time of operation, which I will be happy to furnish if requested.

